# Nitrergic neurons of the dorsal raphe nucleus encode information about stress duration

**DOI:** 10.1371/journal.pone.0187071

**Published:** 2017-11-10

**Authors:** India S. Nichols, Mary I. Jones, Chuma Okere, Godwin Ananaba, Brittany Bush, Cloe Gray, Allison Brager, J. Christopher Ehlen, Ketema Paul

**Affiliations:** 1 Department of Biological Sciences, Clark Atlanta University, Atlanta, Georgia, United States of America; 2 Department of Neurobiology, Morehouse School of Medicine, Atlanta, Georgia, United States of America; Technion Israel Institute of Technology, ISRAEL

## Abstract

Nitrergic neurons of the dorsal raphe nucleus (DRN) may play a role in physiological stress responses. The caudal lateral wings (CLW) are unique compared to other rostral-caudal DRN sub-regions because they contain distinct nitric oxide (NO) synthase (NOS) populations that are independent of tryptophan hydroxylase (TPH). NOS neurons in the CLW are also highly activated during acute restraint stress. However, the effects of acute stress duration on NOS activation in the CLW are unclear. Here NADPH-d, an index of NOS activity, is used to show that sub-regions of the DRN have differential NOS activation in response to 6 hours of restraint stress in rats. We report increased NOS activity through 6 hours of restraint in the caudal lateral wings and ventromedial sub-regions. These data suggest that, NOS neurons may play a dynamic role in the response to stress duration.

## Introduction

**Fig 1:**
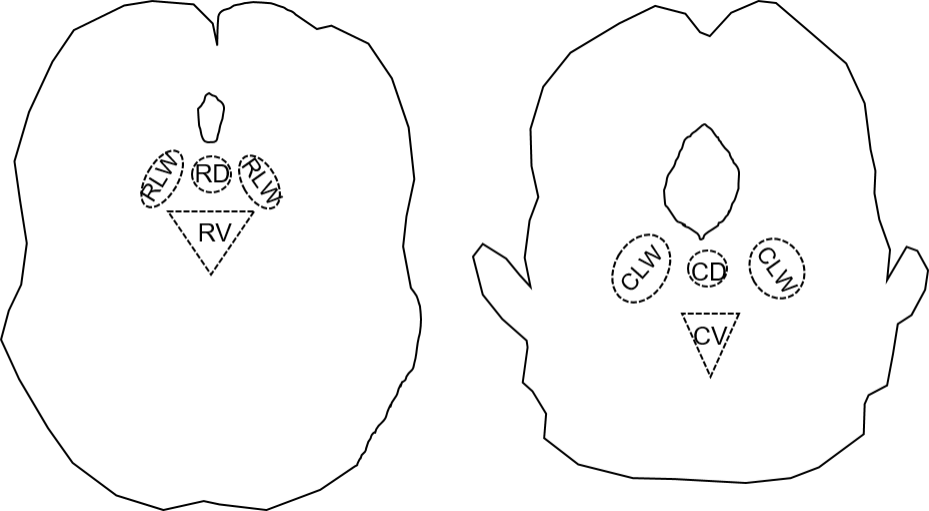
Schematic drawing of the rostral and caudal dorsal raphe nucleus. A. Drawing represents the rostral extension of the dorsal raphe nucleus at Bregma -7.33mm. B. Drawing represents the caudal extension of the dorsal raphe at Bregma -8.33mm. Below the aqueduct is the dorsal region, on each side of the dorsal region are the lateral wings, and below the dorsal region is the ventral region.

The sub-regions of the dorsal raphe nucleus (DRN) are functionally organized based on the localization pattern of serotonin (5-HT) producing neurons. Serotonergic neurons in the DRN are found in rostro-caudal and mediolateral dimensions where each rostral or caudal region contains a dorsomedial, ventromedial, and bilateral (lateral wings) sub-region (Fig 1) [1].

There is a large non-serotonergic domain in the DRN, which includes nitric oxide (NO) synthesizing neurons [1]. Similar to serotonergic neurons, neuronal nitric oxide synthase (NOS) neurons manifest in a unique topographical distribution and co-localization pattern across the DRN [2]. Most of the neurons in the dorsomedial and ventromedial DRN, collectively known as the midline, display both NOS and tryptophan hydroxylase (TPH), but neurons in the caudal lateral wing (CLW) display either NOS or TPH exclusively [2, 3]. Interestingly, more than 75% of the neurons in the CLW that contain NOS were also positive for inhibitory 5-HT_1A_ receptors, suggesting that those neurons are regulated by 5-HT [1, 3, 4]. Furthermore, it has been revealed that NOS neurons in the CLW also co-localize with cholinergic neurons making them a unique subset of neurons in the DRN [4]. The organization of NOS neurons in the DRN may be functionally relevant to the role of the nitrergic system in the stress response. This is because different subsets of neurons in the DRN are involved in different processes and project to different brain regions [5]. Neurons in the lateral wings only project to subcortical structures while neurons from both rostral and caudal regions project to the cortex [2].

Axonal varicosities produce NO in the DRN. Varicosities are commonly distributed in an en passant, “beaded-string” manner along the length of an axon [6]. Varicosities allow for pre-release of neurotransmitters without being coupled to frequency-coded neuronal activity [7]. Neurotransmitters released from varicosities can diffuse over a large distance, in the case of NO, a free radical gas, release from multiple varicosities may increase the diffusion distance by increasing the concentration of NO [8].

The distribution pattern of varicosities is an important feature because the mean spacing is a reflection of synaptic density and provides information about the mechanism of synaptogenesis [9]. NO is an ideal candidate for non-synaptic transmission in the nervous system since its effects are concentration dependent and it has a short half-life [8, 10]. Therefore, an understanding of how acute restraint stress alters the density and spacing of nitrergic axon varicosities within the DRN will provide important insights into potential extra-synaptic signaling that may complement canonical synaptic transmission in the DRN.

In rats, it has been revealed that a single episode of restraint is an ideal method to model acute stress and increase corticosterone levels. The trigger for stress comes from the aversive nature of involuntary immobility. Just one hour of restraint is enough to increase anxiety behavior and corticosterone levels in rats (for review see [11]) while three hours of restraint increases NOS activation in the DRN and Periaqueductal gray [12, 13]. Importantly, the degree of corticosterone level elevation depends upon the duration of restraint. Restraint has been shown to increase corticosterone in rats by 60–90% of baseline levels and four hours of restraint has been shown to increase corticosterone levels for a longer period than restraint for one hour [14, 15]. In addition to restraint stress, other acute stressors can activate nitrergic signaling. For example, a ten-minute exposure to a live cat induces NOS activation in up to 50% of cells in some brain regions, including the DRN [16].

While the evidence of NOS’s role in the stress response is expanding, little is known about how the duration of acute stress affects NOS neurons in the DRN. Duration and modality are important variables to consider when discussing the response to stress. Duration especially has been shown to have different effects on behavioral paradigms such as sleep [17]. Duration of acute stress also effects facilitation of the HPA axis where duration effects the intensity [15].

In this study, we examine NOS signaling discretely as an immediate early stress response mechanism with the goal of characterizing the response to acute stress. If this response is significant, then we will be able to target this system to examine the behavioral responses to stress. To characterize the nitrergic response to acute stress, we examined whether NOS producing cells in DRN sub-regions encode important information about stress duration. This goal was based on the hypothesis that NOS in the DRN dynamically signals temporal information about restraint stress. This hypothesis was tested by determining the effect of increasing bouts of acute restraint on the density and intervaricosity spacing of NADPH-d profiles compared to that in freely behaving, control animals.

## Materials and methods

### Ethics

The Morehouse School of Medicine Institutional Animal Care and Use Committee approved all procedures. Therefore, this study was conducted in accordance with recommendations in the Guide for the Care and Use of Laboratory Animals of the National Institutes of Health. Animals were perfused following 3% isoflurane anesthesia.

### Animals

Twenty male Long Evans rats (170–200 grams; Charles River, Wilmington, MA, USA) were maintained on a 12h light/dark cycle (lights on at 9:00am). They were group housed in a standard cage, with four per cage, in a climate-controlled (68°F-79°F, 70°F is the target) environment; food and water were provided *ad libitum*. This strain was selected because of previous studies validating NOS activity following restraint [15, 16]. The sample size of five animals per group is based from previous studies that measured NOS activity in the DRN and dlPAG [12, 13].

### Restraint stress

The animals were group housed (4/cage) prior to being randomly assigned to one of four groups: control, one-hour, three-hours or six-hours of restraint. Restraints were performed in a semi-cylindrical, Plexiglas, restraint device (20 cm x 5.5 cm), one rat per restraint device. On the day of treatment, rats were randomly selected for restraint. Those that were selected for six-hours of restraint were placed in the restraint at the time of lights on, zeitgeber time 0 (ZT0), three-hour rats were placed in the restraint three hours later,at ZT3, and 1-hour restraint rats were placed in the restraint at ZT5. These time points were selected based on the circadian variation of NOS activity in the brain [18]. The restraints were placed in secure areas in the laboratory. Control rats were maintained in their home cage with *ad libitum* access to food and water. The criteria for removal from restraint device were the presence of lesions, illnesses, deleterious or maladaptive behavior, or other behavioral changes. No animals needed to be removed from restraints.

### Tissue collection

Immediately following restraint and under deep isoflurane anesthesia, rats were perfused transcardially with ice-cold 0.1 M phosphate buffered saline (PBS) followed by cold paraformaldehyde (4% in 0.1 M PBS). The brains were removed and post-fixed overnight in paraformaldehyde (4% in 0.1 M PBS) at 4° C then transferred to 30% sucrose. Coronal sections (20 μm) starting from -7.33 mm relative to bregma through the DRN neuraxis were collected. The settings on the cryostat were Fine: 10, Trim 20, and temperature -20°F. The sections were collected based on the rat dorsal raphe nucleus figures from “Anatomic and Functional Topography of the DRN” by Abrams. From -7.33 to -7.64 rostral and -8.54 to -9.26 caudal.

### NADPH-d for NOS activation

Brain sections were washed 3x with PBS in a 24 well plate; floating sections were stained with NADPH-d (incubated in 0.1 M PBS, pH 7.4, containing 0.3% Triton-X-100, 0.1 mg/ml nitroblue tetrazolium and 1.0 mg/ml NADPH) overnight in a 37°C incubator. Following incubation, the sections were washed 3x at 5 minutes each in PBS and mounted on gelatin-subbed microscope slides. The sections were dried overnight in a slide drawer then cleared and dehydrated with alcohol before cover slips were applied.

### Imaging

Immunohistochemical analysis was performed on six sub-regions of the DRN defined using stereotaxic coordinates. In the rostro-caudal dimension: rostral (-7.3 to -7.73 mm from Bregma and caudal -8.45 to -9.26mm Bregma). Analysis was performed on every fourth coronal section. A Zeiss microscope fitted for AxioCamMR3 for grayscale and Axio imaging software was used to capture all images. All the images were taken under an ‘auto exposure’ function of the imaging system to minimize contrast bias.

### Image processing

All images were converted from RGB color to gray scale using ImageJ. Three sections for each rostral-caudal sub-region was used to obtain NADPH-d density and somatic cell count.

Optical density (OD) was obtained by measuring the mean gray pixel value from representative neurons in each sub region with Image J software. Then using the calibrated OD curve to obtain the actual OD value. The mean gray value is the sum of the gray values of all the pixels in the selection divided by the number of pixels. Image J uses mean gray value as a measure of optical density.

### Intervaricosity distance measurements

Intervaricosity distances were obtained on axons with at least three varicosities. The distances were measured using the ImageJ straight-line tool. A straight line was placed between two varicosities and automatically calculated by the software in pixels. To determine the length in micrometers the ImageJ scale was set from a known length of distance obtained from the Axiovision software.

### Statistical analysis

SPSS software was used for statistical analysis. Repeated Measures ANOVA was used to determine the between effect of duration and within effect of sub-region on NOS neuron counts, NOS activation, and, intervaricosity distance with significance being determined by a p-value less than 0.05. Levene’s test of equality of error variances was used to determine homogeneity and due to unequal variances Dunnet’s T3 comparison was used as a post hoc to compare each duration against the control. Percentage change was calculated in excel by subtracting the mean from each animal to the mean of the control group then dividing by the mean of the control and multiplying by 100. Graphs were generated on excel and error bars are representative of standard error.

## Results

There were no signs of health issues immediately after the rats were removed from the restraints. One rat was omitted from the 1-hour group because it escaped from the restraint device. See Table 1 for a summary of the results and all data is available in the S1 Fig file.

**Table 1:** Summary table of NOS activity in the DRN.

	Rostral	Caudal
Dorsomedial	- Low number of NADPH-d neurons overall but higher than caudal dorsomedial - No varicose axons - NADPH-d staining similar to Rostral Ventromedial	- Low number of NADPH-d neurons and less NADPH-d staining compared to rostral dorsomedial - Very few varicose axons
Ventromedial	- High number of NADPH-d neurons and intense NADPH-d staining compared to other sub-regions except caudal lateral wings - 3–6 hours of restraint increases NADPH-d staining - Intervaricosity distance is lower than lateral wings but similar to caudal ventromedial. - 1–3 hours of restraint decreased intervaricosity distance	- Moderate number of neurons, lower than rostral ventromedial and caudal lateral wing but higher than rostral dorsomedial and rostral lateral wing - 6 hours of restraint increases NADPH-d staining - Inter-varicosity distance is lower than lateral wings but similar to rostral ventromedial. 1–6 hours of restraint decreases intervaricosity distance
Lateral Wing	- Very low number of NADPH-d neurons and NADPH-d staining - No varicose axons	- High number of NADPH-d neurons and intense NADPH-d staining compared to other subregions except rostral ventromedial - 6 hours of restraint increases NADPH-d staining - Intervaricosity distance is the highest - 1–3 hours of restraint induces a decrease in intervaricosity distance

### Sub-regional effect of NOS activity

**Fig 2:**
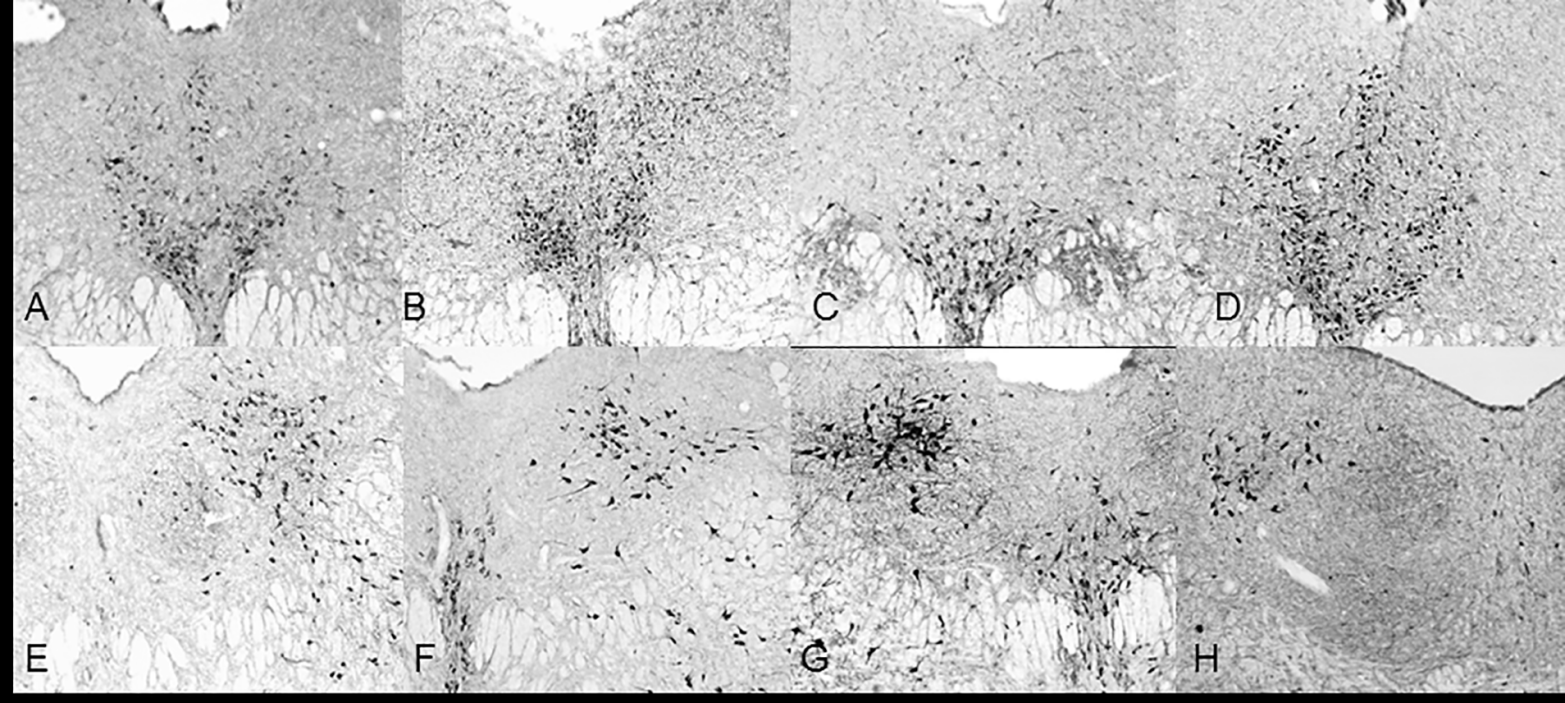
5x objective NADPH-diaphorase staining in the DRN. Rostral (A, B, C,D) and caudal (E, F, G, H) halves of the DRN in control (A, E), 1 hour (B, F), 3 hour (C, G) and 6 hour (D, H) restraint rats. These photo images are rostral (Bregma -7.64mm) and caudal (-Bregma -8.36) extensions of the DRN through a 6 hour restraint duration. The blue color formed by NADPH-d reaction was adjusted to gray scale for contrast and each image was adjusted against background staining.

**Fig 3:**
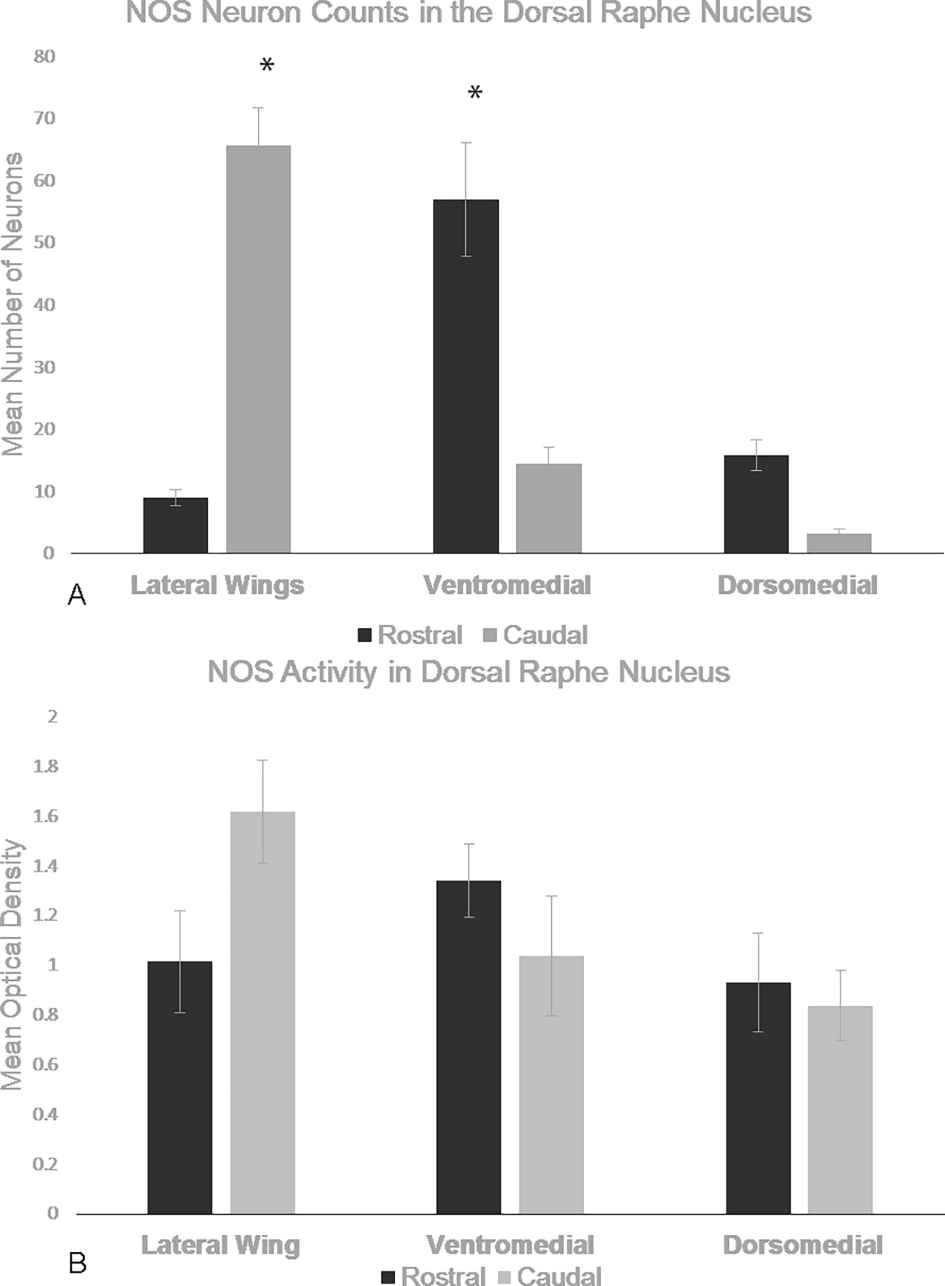
Caudal lateral wings and rostral ventromedial have most NOS activation. These graph shows mean levels of NADPH-d stained neurons in control animals for each DRN sub-region. The CLW and RV areas have the most NADPH-d staining compared to other regions of the DRN. Error bars are representative of standard error. Significance is denoted by an asterisk and these regions are compared to every other region of the DRN.

There was a sub-regional effect on the number of NADPH-d stained neurons in the DRN (F_5, 75_) = 186.218, *p*<0.001, Repeated-Measures ANOVA). NADPH-d staining was mostly localized to the rostral ventromedial and caudal lateral wing sub-regions (Fig 2 and Fig 3, respectively). These regions contained the highest number of neurons (n = 5, 57±20 and 66±13, respectively) compared to the rostral lateral wings (9±3, Fig 2 and Fig 3), rostral dorsomedial (16±6), caudal dorsomedial (3±2), and caudal ventromedial (14±6,) regions.

Optical density measurements did not show significant sub-regional effect (F_5,70_ = 2.578, *p* = 0.077, Repeated Measures) on NADPH-d staining intensity but there was a trend that showed the rostral ventromedial (n = 5, 1.34OD± 0.33) and rostral dorsal (n = 5, 0.933OD ±0.44) regions had more intense staining than their caudal counterparts (n = 5 CV = 1.04OD±0.48, CD = 0.84OD±0.28) while the caudal lateral wings (n = 5, 1.62OD±0.27) had more intense staining than the rostral lateral wings (n = 5, 1.02OD±0.41).

**Fig 4:**
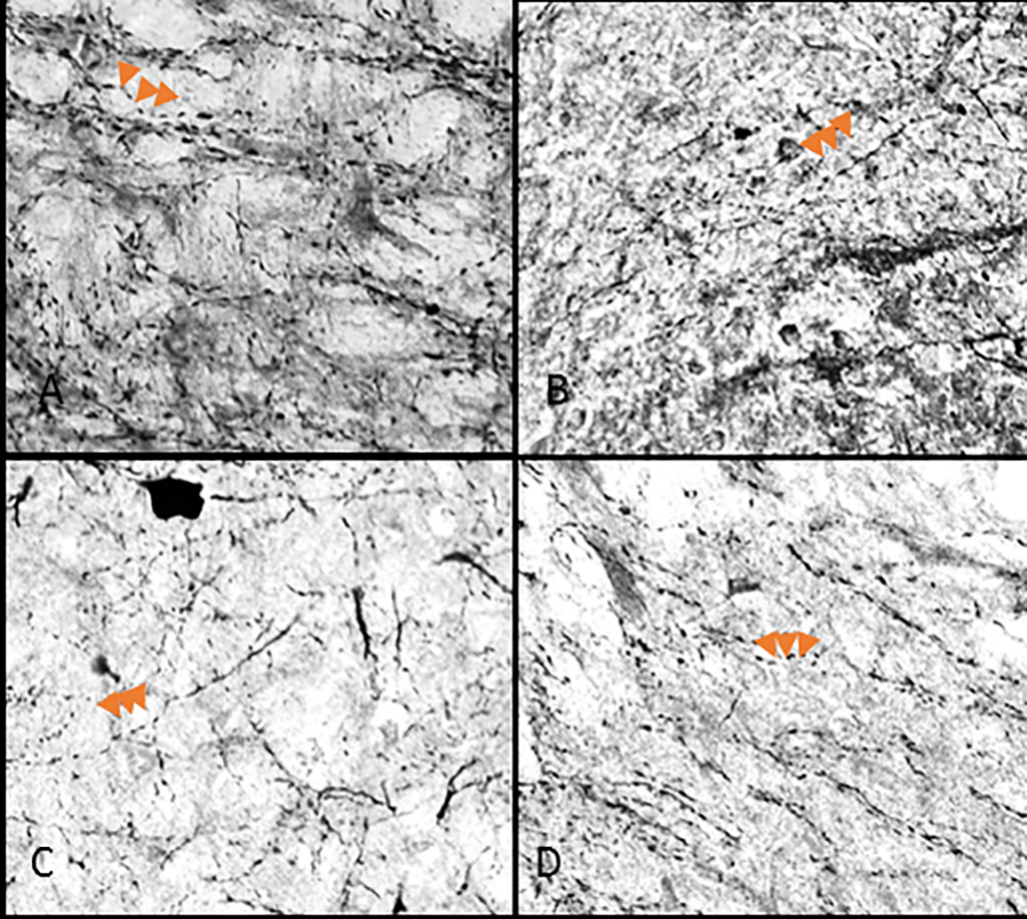
40x objective varicosity fibers in the caudal lateral wings. Caudal extension of the DRN in control (A,), 1 hour (B,), 3 hour (C,) and 6 hour (D,) restraint rats. Arrows point to three varicose structures on axons.

**Fig 5:**
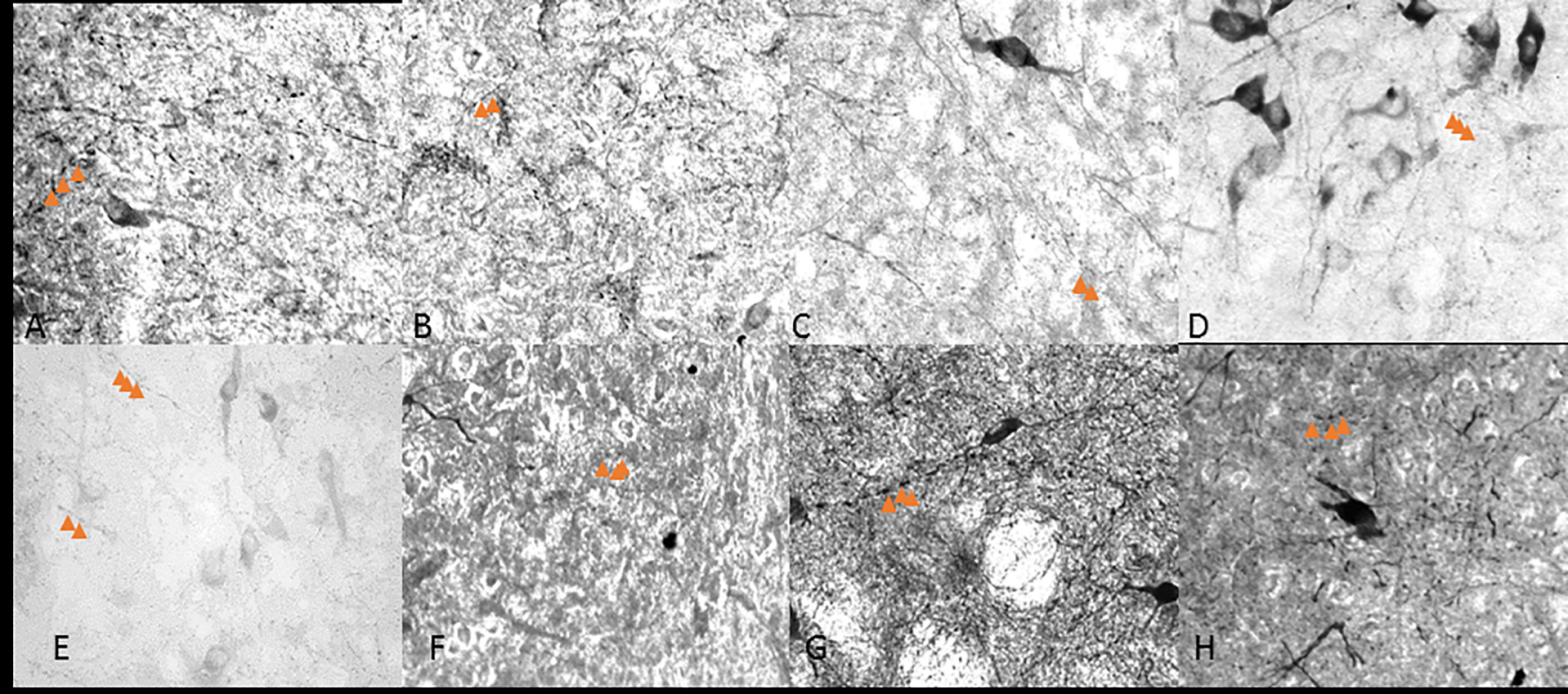
40x objective varicosity fibers in the ventromedial. Rostral (A,B,C,D) and caudal (E,F,G,H) halves of the DRN in control (A,E), 1 hour (B,F), 3 hour (C,G) and 6 hour (D,H) restraint rats. Arrows point to varicose structures on axons. Compared to the images in Fig 3, these figures have less abundant varicose axons.

The rostral lateral wings and each dorsal region had few to zero axons with varicosities so, those regions were omitted from intervaricosity analysis. Intervaricosity distance showed significant differences depending on sub-region (F_2,24_ = 11.192, _*p*_<0.001). In particular, the caudal lateral wings (9.30μm± 1.57, Fig 4) had higher intervaricosity distances compared to both rostral (6.06μm ±0.76 Fig 5) and caudal (6.42μm ± 0.97) ventromedial regions.

### Duration effect of NOS activity

**Fig 6:**
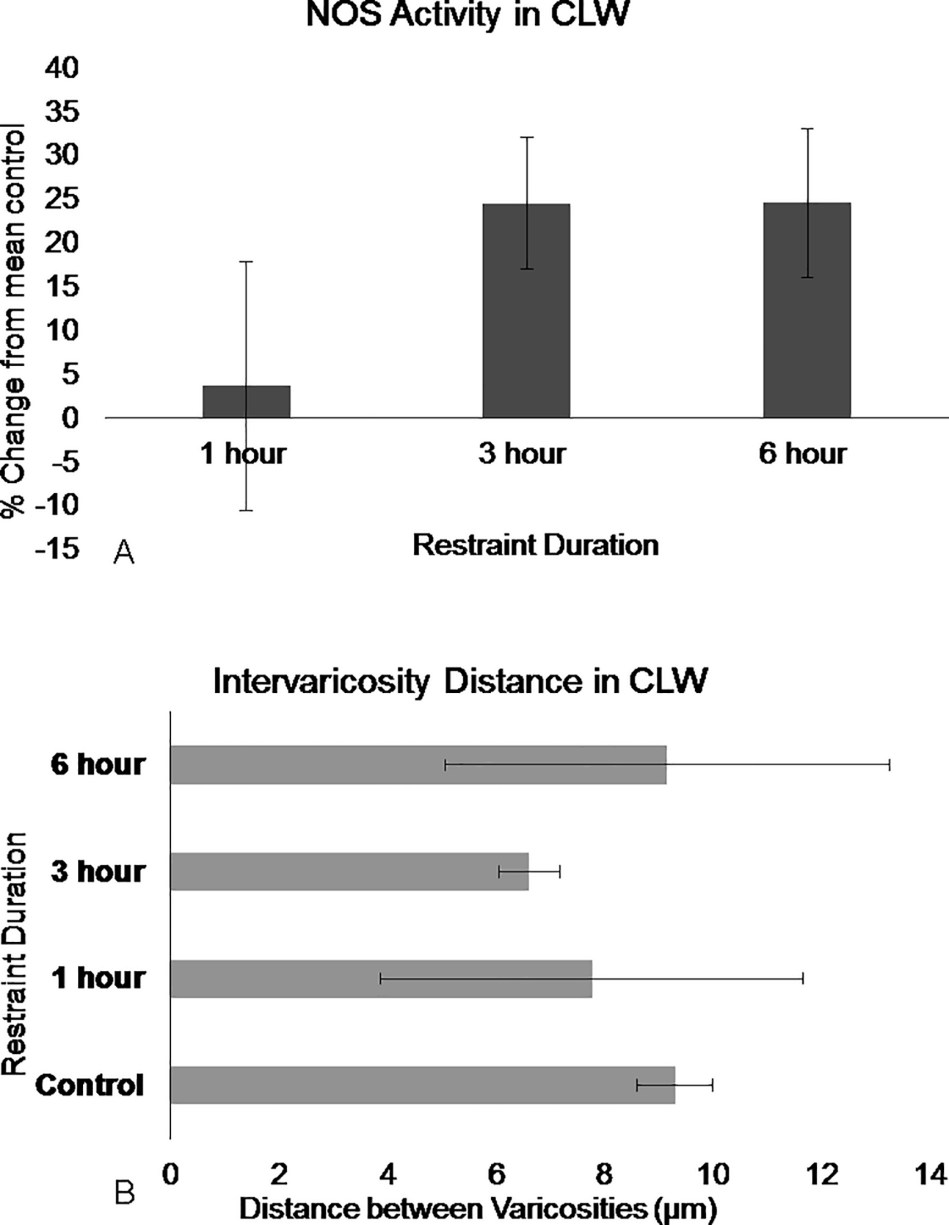
NOS in the DRN caudal lateral wings show differences in activation following restraint stress. (A) NOS activation increase in the caudal lateral wings region following 3 hours and significantly following 6 hours of restraint. (B) Intervaricosity spacing decreases following 3 hours of restraint but by 6 hours, the intervaricosity spacing is closer to control values. Percentages were calculated by subtracting each animal per experimental group by the mean of the control then dividing by the control mean and multiplying by 100. Significance is denoted by an asterisk and compared to the control. Error bars are representative of standard error.

**Fig 7:**
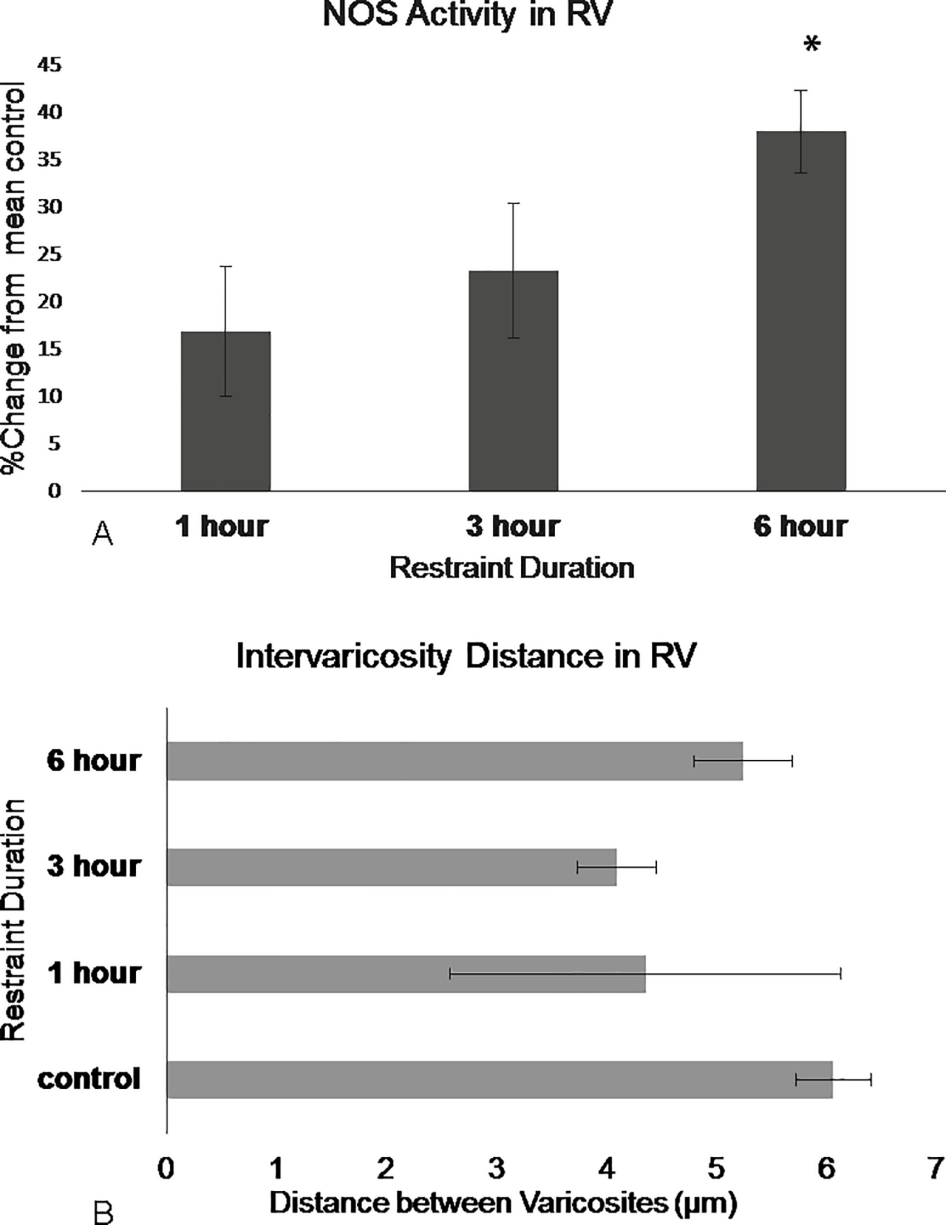
NOS in the DRN rostral ventromedial region show differences in activation following restraint stress. (A) NOS activity barely shifts after 1 hour of restraint then increases following 3 hours of restraint and significantly increases following 6 hours of restraint. (B) Intervaricosity spacing decreases following 3 hours of restraint but by 6 hours, the intervaricosity spacing is closer to control values. Percentages were calculated by subtracting each animal per experimental group by the mean of the control then dividing by the control mean and multiplying by 100. Significance is denoted by an asterisk and compared to the control. Error bars are representative of standard error.

**Fig 8:**
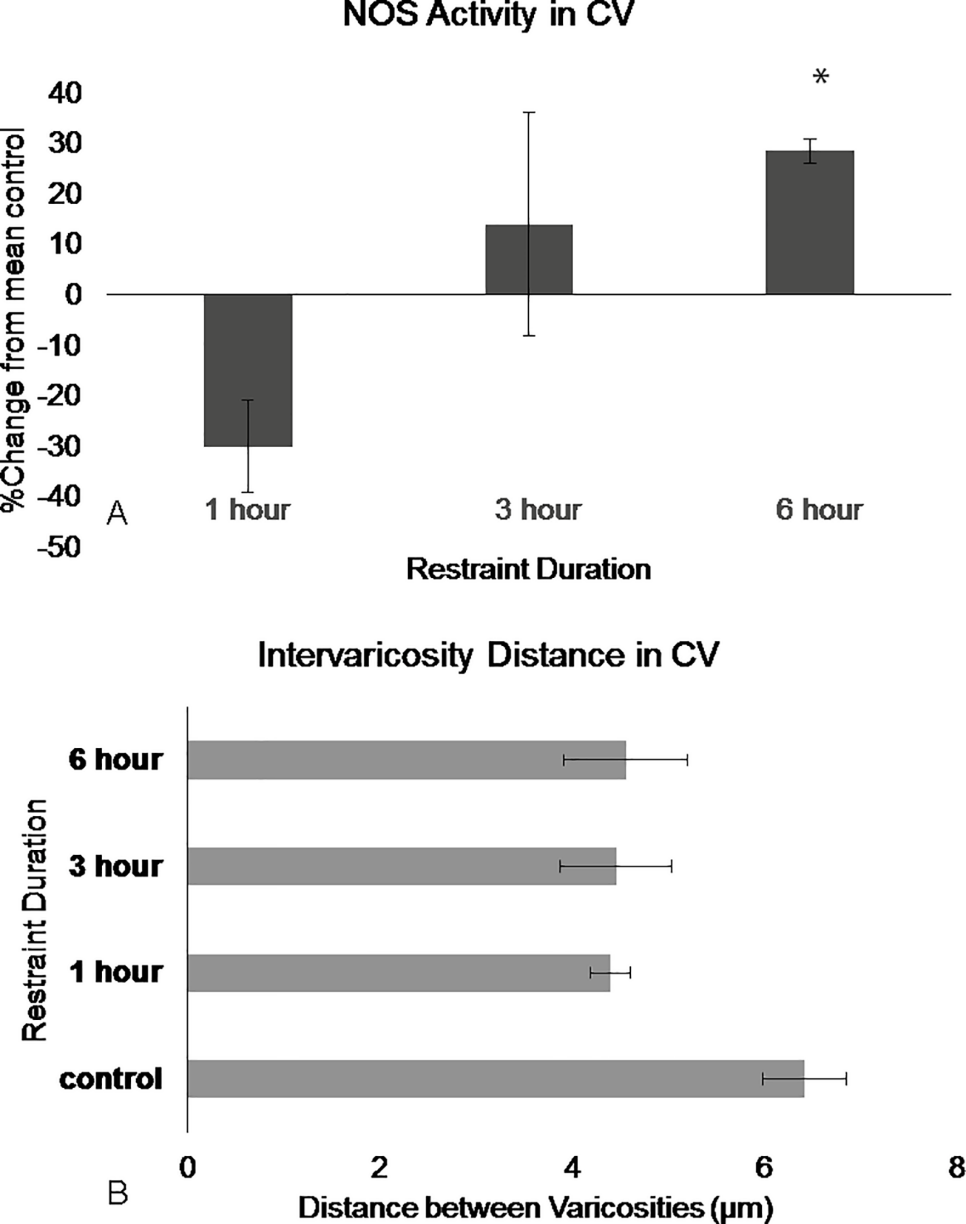
NOS in the DRN caudal ventromedial region show differences in activation following restraint stress. (A) NOS activity in the caudal ventromedial remains stable following 3 hours of restraint but significantly, increase following 6 hours of restraint. (B) Intervaricosity distance decreases following 1 hour of restraint and remains decreased through 6 hours of restraint. Percentages were calculated by subtracting each animal per experimental group by the mean of the control then dividing by the control mean and multiplying by 100. Significance is denoted by an asterisk and compared to the control. Error bars are representative of standard error.

Restraint stress had no significant effect on the number of NADPH-d stained neurons (F_3,15_ = 0.273, p = 0.844, Repeated Measures ANOVA). However, restraint duration had an effect of NADPH-d intensity (F_3,15_ = 5.501, p = 0.009 Repeated Measures ANOVA). The Levene’s test revealed non-homogenous variances in the RV and CV sub-regions (p>0.05 F_3,15_ = 3.484 and F_3,15_ = 11.472, respectively). Within each rostral and caudal sub-region, there was no significant effect following one-hour or three-hours of restraint (p = n.s, Dunnet’s T3). Dunnet’s T3 post hoc test revealed differences between the control animals and animals restrained for six-hours (p = 0.018), There was 25% ±19 increase in the caudal lateral wings (Fig 6), 38% ±9.5 increase in the rostral ventromedial region (Fig 7), and 28%± 5.3 increase in the caudal ventromedial regions (Fig 8, S2 Fig).

The effect duration had on intervaricosity distance was not significant in the three sub-regions but there were obvious trends. (F_3, 12_ = 2.620, p = 0.099, Repeated Measures ANOVA). Most animals in the one-hour restraint group showed decreased intervaricosity distances compared to control animals in the rostral ventromedial (4.39μm ± 3.56, Fig 7), caudal ventromedial (4.46μm ± 1.30), and caudal lateral wings (7.76 μm ± 1.84, Fig 6). Three-hours of restraint also showed decreased intervaricosity distance in the rostral ventromedial (4.10μm ± 0.80, Fig 7), caudal lateral wings (6.61μm ± 1.27, Fig 6), and caudal ventral regions (4.45μm ± 1.30, Fig 8) compared to control animals (6.69μm ± 0.44, 9.29μm ± 1.57, and 6.12μm ± 1.15, respectively). Interestingly, Six-hours of restraint showed an increased intervaricosity distance compared to one-hour and three-hour restraint animals in the caudal lateral wings and rostral ventromedial (5.24 μm ± 0.99 and 9.14 μm ± 2.18, Figs 6 and 7, respectively) but intervaricosity distance in the caudal ventral region remained decreased compared to the control group (4.56 μm ± 1.43).

## Discussion

Study of the role NOS plays in the stress response is important because it has been reported that NOS activation in the DRN has anxiolytic-like and antidepressant-like effects [19]. Here, we have substantiated that the DRN is a heterogeneous structure by showing the localization and activation of NOS following restraint stress. There is a disproportionate activation pattern of NOS, where the CLW and RV have the highest number of NOS-containing neurons compared to other sub-regions (Fig 5). We also show that NOS has a dynamic temporal response to acute stress through 6 hours of restraint. There was no significant change in activity following one-hour of restraint nor three-hours of restraint however following 6 hours of restraint, there is significant increase in NOS activity as measured by the intensity staining of NADPH-d.

This study expands on a 2006 study by Okere and Waterhouse [12], which examined NOS activation in the DRN following 3 hours of restraint using NADPH-d to localize NOS activity. NADPH-d was also used in the current study to observe the effects that up to 6 hours of acute restraint would have on nNOS activity profile. The NADPH-d assay is well documented to localize and measure activity-dependent changes in NOS producing neurons; therefore, NADPH-d staining refers to NOS activity [20, 21, 22, 23]. Similar to Okere’s findings we observed increased NOS activation in the DRN immediately following 3 hours of acute restraint. More specifically, there was more activation in the RVM and CLW than any other sub-region, which was enhanced by restraint stress. We also observed increase in both the total rostral and total caudal sub-regions, especially following 6 hours of restraint. Since there was not a significant difference in the number of neurons expressing NOS, this data suggests that acute restraint encodes data about stress duration by altering the activity levels of NOS neurons.

**Fig 9:**
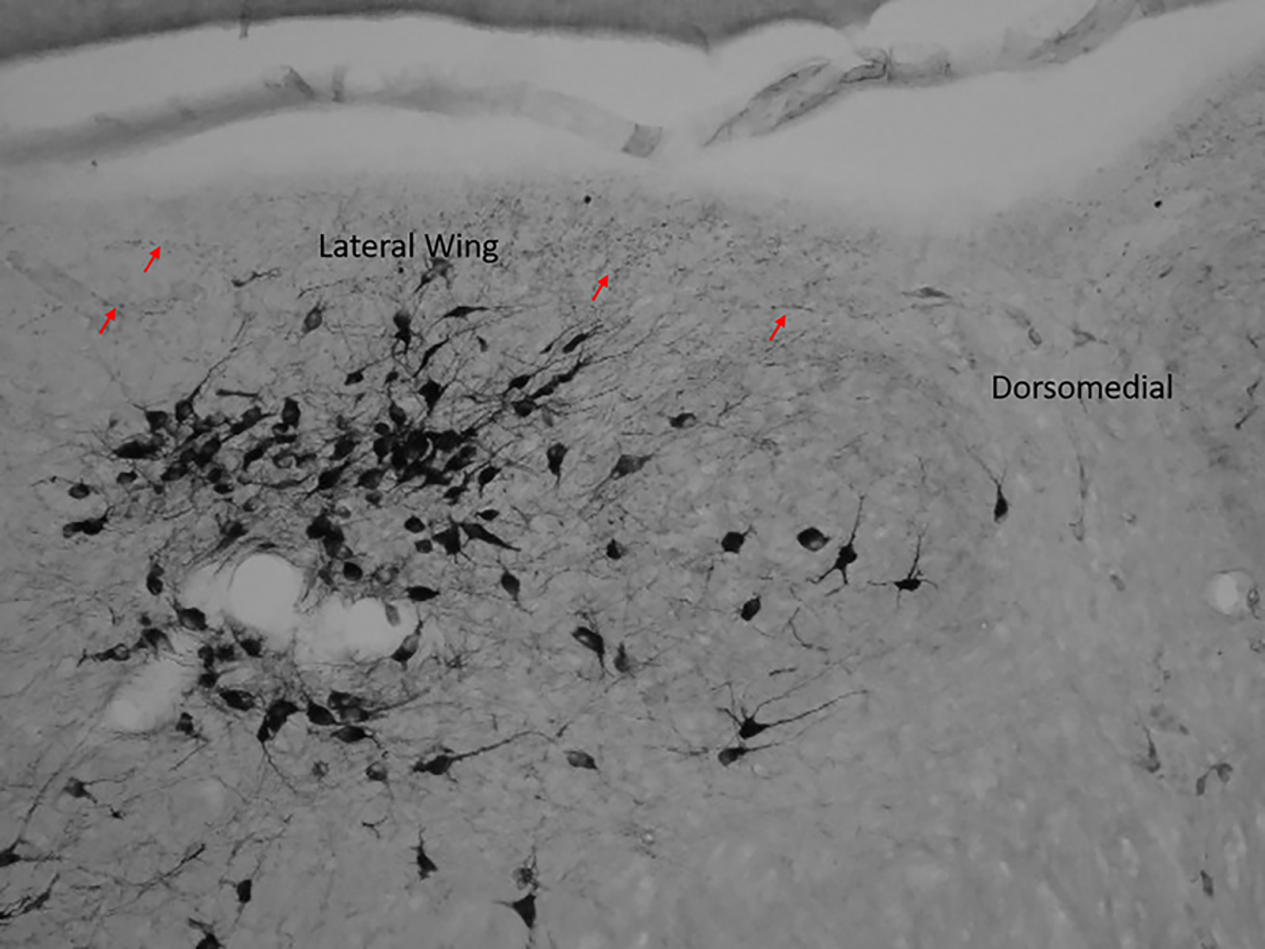
Varicose axon localization in the caudal lateral wing. The red arrows indicate varicose axons surrounding the aqueduct in the lateral wing of the caudal sub-region. The cluster of neural bodies does not contain many varicose axons.

We added another level of analysis by examining the varicosities and intervaricosity distance in response to acute stress. The study of varicosities is important because mean spacing of intervaricosity distance reflects synaptic density and therefore gives insight to neural signaling during restraint stress [6, 9]. Studying the release method of nitric oxide is important because it is immediately released via diffusion as soon as it is produced and it has a short half-life. Theoretical models have suggested that when NO is released from a multiple sources in close proximity, such as varicosities, it increases the concentration of the gaseous transmitter [8]. Furthermore, this analysis is crucial for drug discovery because it provides a better understanding of the sources of NO release. Previous studies have also shown that 3 hours of restraint stress decreases intervaricosity distance in NOS axons of the dlPAG, while increasing both the density of axonal varicosities, and NOS activation, immediately after exposure to restraint. [13]. In our study, we demonstrated decreased intervaricosity spacing in the caudal lateral wing, rostral ventromedial, and caudal ventromedial of rats restrained from one to three hours. It is important to note that the location of varicosities were mostly in-between sub-regions (Fig 9). In Fig 9, the varicosities mostly line the aqueduct of the caudal lateral wing sub-region and there are few varicosities noticeable within the cluster of neural somas. We also observed differences in axon morphology types in the rats that were restrained compared to unrestrained rats (Fig 3). There were thinner fibers with decreased intervaricosity spacing in restrained rats compared to thick fibers in control rats. NOS neurons that project to the DRN include many regions involved in the stress response. Specifically, the paraventricular nucleus of the hypothalamus projects to both ventromedial and lateral wing regions [2]. The activation of thinner fibers in the lateral wings began at 1 hour of restraint and continued through 3 hours of restraint, which suggest that signaling from other regions occurs before increased NOS activation from DRN neurons (Figs 3, 4, 6B and 7B). With the origin differences of NOS fibers afferent to the midline areas and lateral wings and the functional heterogeneity of DRN neuronal populations, these observations suggest an underlying physiological significance of the unique distribution of NOS in the DRN that has implications for nitrergic pathways involved in stress.

Taken together, these results suggest that NOS cells in the DRN are dynamic in their response to acute restraint stress and this dynamic response is both dependent upon duration of the applied stressor and location in the DRN. These results corroborate substantial evidence that sub-regional activation is a hallmark of the DRN, as activation of each region is associated with different behavioral outcomes or stimuli. For example, activation of neurons in mid-caudal and caudal regions are associated with anxiety processing, while lateral wing neurons are responsive to panic-evoking situations [5]. These differential responses are probably related to the fact that DRN sub-regions project to distinct regions as demonstrated when the forced swim test increased extracellular levels of 5-HT in the striatum at the same time extracellular levels of 5-HT was decreased in the lateral septum [24]. Those differences were due to distinct subpopulations of DRN projections where caudal neurons projected to the lateral septum and the striatum received more projections from the rostral region [21]. Since the DRN projects to both cortical and sub-cortical structures, the activation of NOS during stress is interesting because it suggests a role for NOS in the DRN during the stress response.

Restraint duration is an important factor to consider when studying stress response because it has an influence on behavior. Evidence shows that duration of restraint stress has a direct effect on rapid eye movement (REM) and non-rapid eye movement (NREM) types of sleep [17]. For instance, 0.5 hours of restraint showed increases in both REM and NREM while 4 hours of restraint showed no changes in either REM or NREM [17]. Therefore, it is evident that the duration of restraint influences the behavioral response to stress. Given what our experiments revealed about NOS activation as it relates to stress duration, future studies need to be conducted to explore how these differences in NOS activation influence behavioral outcomes and sleep architecture.

In conclusion, NOS in the DRN undergoes many changes through a 6-hour duration of stress. These studies show not only changes in the cell bodies but also the response in axons projecting to the region. This study suggests that NOS in the DRN is responsible for encoding information about prolonged stress and there may be a nitrergic pathway involved in the response to stress. In future studies, we would like to explore the regulation of NOS by 5-HT_1A_ activation and/or 5-HT release especially in the CLW region.

### Technical considerations

In our study, we used isoflurane as an anesthetic. Despite reports that isoflurane may regulate NOS signaling we were able to obtain significant results across DRN sub-regions similar to previous reports using Nembutal as an anesthetic [25, 12].

While in some brain regions, NADPH-d activity is not exclusively due to the neuronal NOS isoform such as the dlPAG, in the DRN it has been verified that NADPH-d is only found in nNOS containing cells [4, 12, 21, 23]. It has also been demonstrated that cellular intensity of NADPH-d is subject to experimental manipulation and the amount of formazan blue produced is a reflection of NOS activity at the time of fixation [4, 12]. Changes in fixation and tissue processing parameters may cause variations in immunohistochemical association of NADPH-d intensity with NOS the experimental and control animals were simultaneously processed under identical conditions throughout restraint handling, isoflurane exposure, and tissue preparation to reduce inter-staining bias to the minimum.

## Supporting information

S1 Fig Complete data for each sub-region at each duration. This data table shows the raw data that was used to determine significance.
(XLSX)

S2 Fig Optical density graphs for individual animals. These graphs show the percentage difference for each animal compared to the mean of control animals. The six-hour animals show the most consistent differences across sub-regions, especially in the caudal ventromedial (cv) sub-region.
(XLSX)

S3 Fig NC3Rs ARRIVE guidelines checklist. (DOCX)
