# Supplementary material for: Nitrergic neurons of the dorsal raphe nucleus encode information about stress duration
Source: PLoS One. 2017 Nov 10;12(11):e0187071. doi: 10.1371/journal.pone.0187071 (PMC5681257; doi:10.1371/journal.pone.0187071)

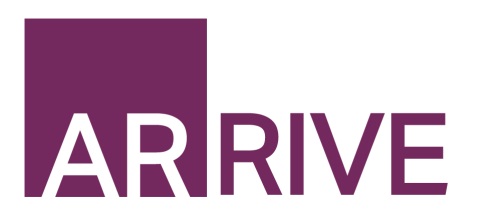


The ARRIVE Guidelines Checklist

Animal Research: Reporting In Vivo Experiments

Carol Kilkenny^1^, William J Browne^2^, Innes C Cuthill^3^, Michael Emerson^4^ and Douglas G Altman^5^

*^1^The National Centre for the Replacement, Refinement and Reduction of Animals in Research, London, UK, ^2^School of Veterinary Science, University of Bristol, Bristol, UK, ^3^School of Biological Sciences, University of Bristol, Bristol, UK, ^4^National Heart and Lung Institute, Imperial College London, UK, ^5^Centre for Statistics in Medicine, University of Oxford, Oxford, UK.*

|  | | ITEM | RECOMMENDATION | Section/ Paragraph |
| --- | --- | --- | --- | --- |
| 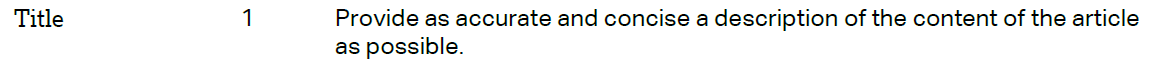 | | | Title Page |  |
| 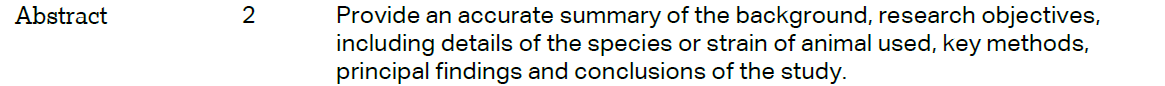 | | | Abstract, page 2 |  |
| INTRODUCTION | | |  |  |
| 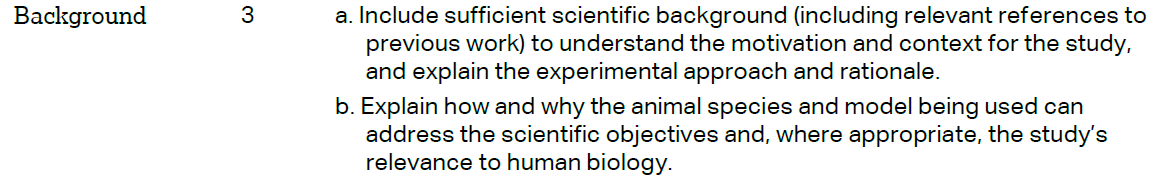 | | | a. page 3-5 b. page 4-5 lines 79-89 |  |
| 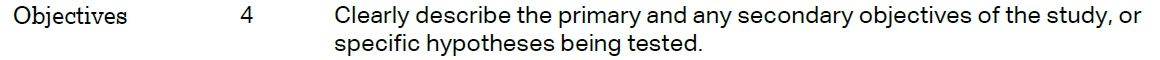 | | | Page 5, lines 96-105 |  |
| METHODS | | |  |  |
| 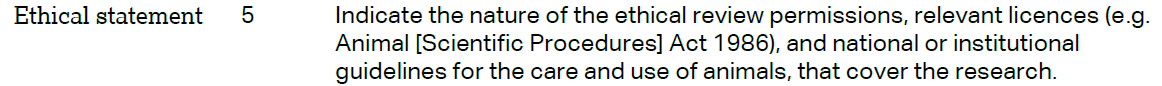 | | | Page 6, lines 111-114 |  |
| 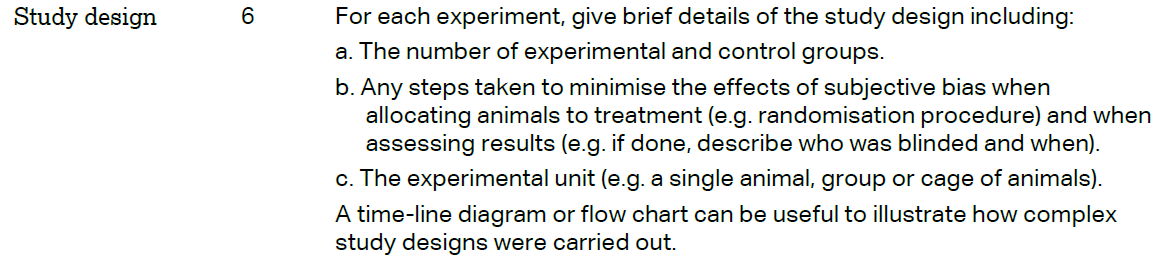 | | | Page 6  a. line 116  b. line 124  c. lines 124-125 |  |
| 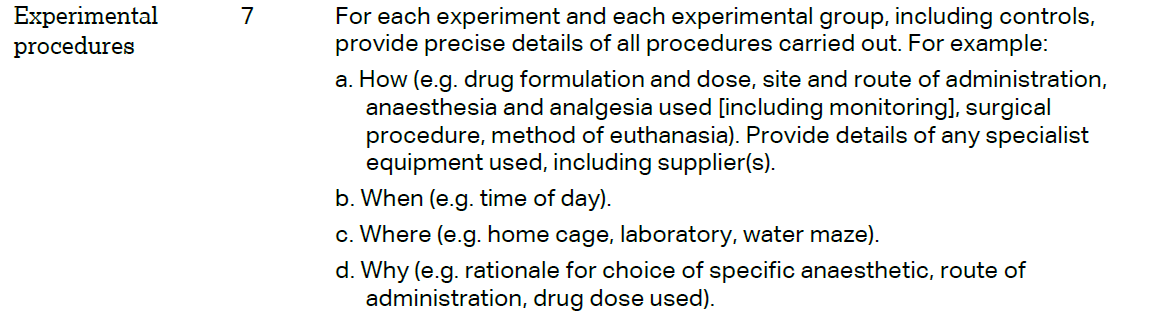 | | | 1. page 6 lines 124-135 2. page 6-7 lines 128-130 3. page 7 line 131 4. page 4 line 79-89 |  |
| 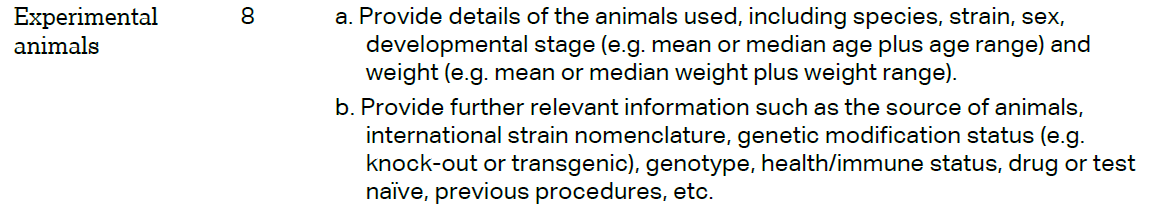 | | | a. page 6 line 116  b. page 6 line 116 |  |

| 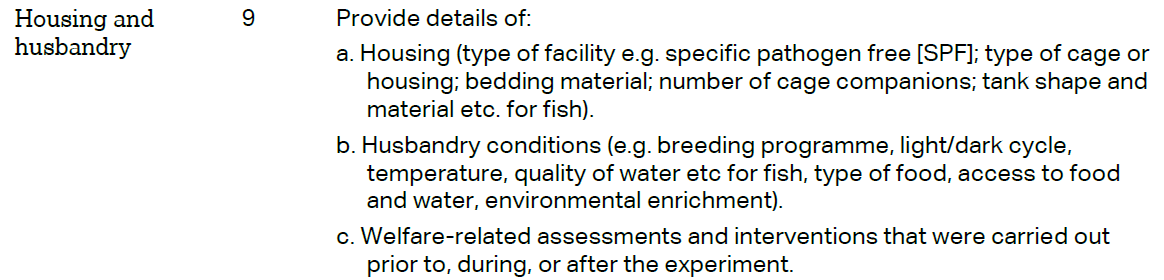 | a. page 6, 117-118  b. page 6, line 117  c. page 7, lines 130-133-135 | |
| --- | --- | --- |
| 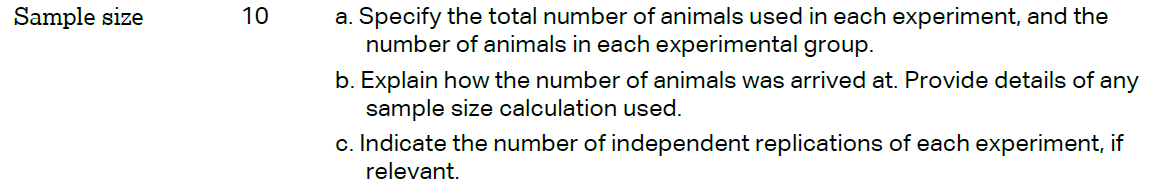 | a. page 6, line 116,  b. page 6, line 121 | |
| 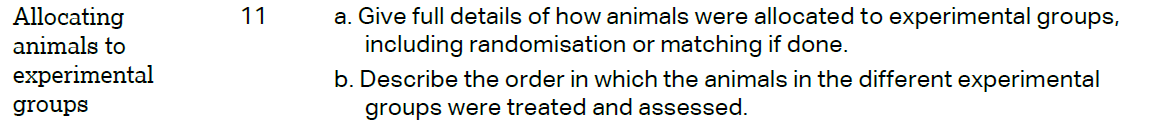 | a. page 6, line 127  b. page 6-7, lines 127-131 | |
| 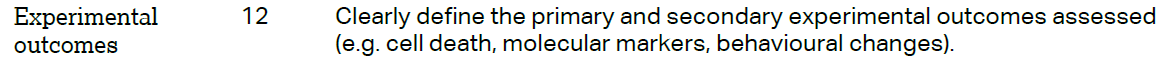 | Page 8, line 154-174 | |
| 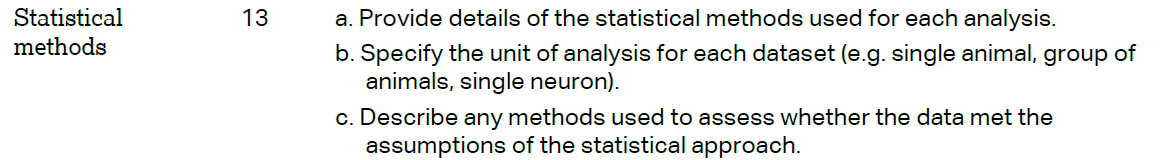 | a. Page 9, lines 176-184  b. page 9, line 177-178 | |
| RESULTS |  | |
| 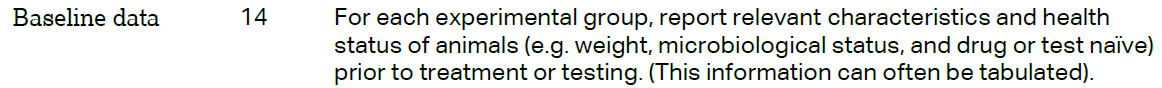 | Page 10, line 188-190 | |
| 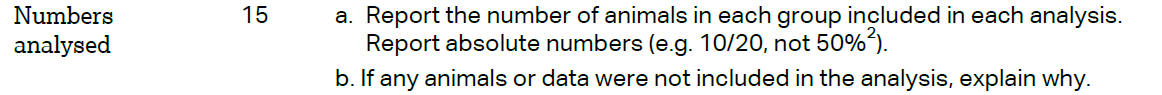 | This is done throughout the results section starting page 10, line 195 | |
| 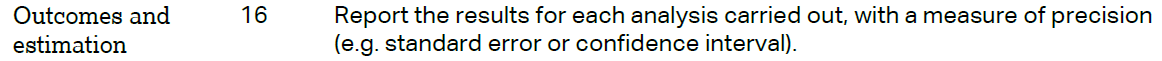 | Results section page 10-12 | |
| 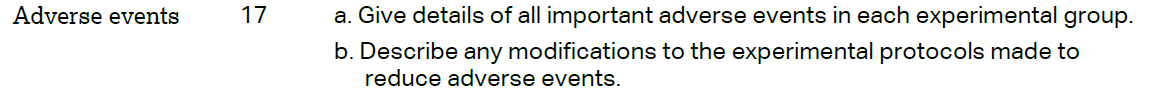 | None to report | |
| DISCUSSION |  | |
| 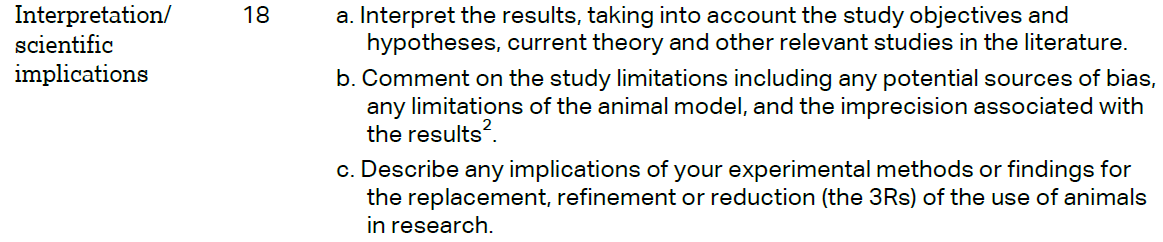 | a. page 13-16  b. page 17, line 321  c. N/A | |
| 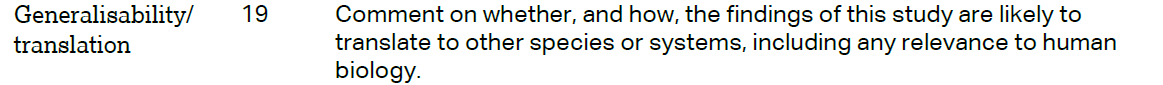 | The relevance to behaviour is in the discussion section | |
| 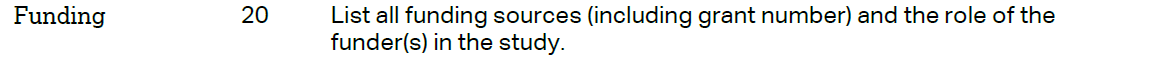 | | Pg 17, Line 340 |

The ARRIVE guidelines. Originally published in *PLoS Biology*, June 2010^1^


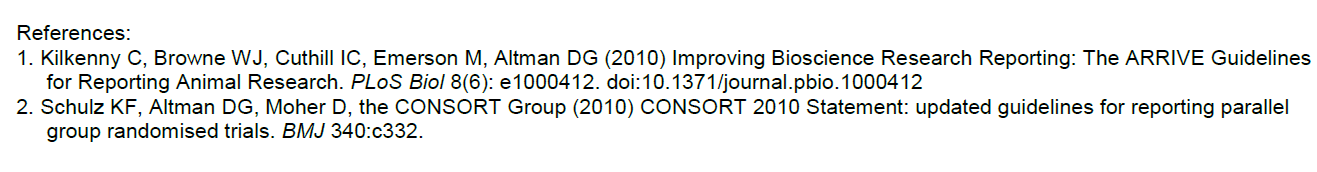

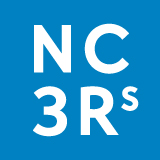

Supplement: S3 Fig — (DOCX) [file pone.0187071.s003.docx]
